# Prognostic Effect of Sarcopenia and Nutritional Factors on Survival in Patients With Newly Diagnosed Multiple Myeloma: A Retrospective Exploratory Study

**DOI:** 10.1002/jcsm.70271

**Published:** 2026-03-28

**Authors:** Xuxing Shen, Jiapei Yu, Xin Cheng, Ye Yao, Yuanyuan Jin, Qinglin Shi, Lijuan Chen

**Affiliations:** ^1^ Department of Hematology The First Affiliated Hospital of Nanjing Medical University, Jiangsu Province Hospital Nanjing China

**Keywords:** multiple myeloma, nutritional assessments, prognosis, sarcopenia

## Abstract

**Background:**

Multiple myeloma (MM) is a hematological malignancy associated with poor prognosis. Prognostic prediction based on patient fitness may facilitate the optimization of therapeutic strategies. This study aimed to explore the prognostic significance of sarcopenia, the prognostic nutritional index (PNI) and the controlling nutritional status (CONUT) on the survival of patients with MM.

**Methods:**

A retrospective analysis was conducted on patients with newly diagnosed MM (NDMM) at Jiangsu Province Hospital between January 2016 and December 2022. Skeletal muscle mass was measured using CT images at diagnosis and normalized for stature to calculate the skeletal muscle index (SMI). Patients with low SMI were defined as sarcopenic. Thresholds for sarcopenia, PNI and CONUT score were determined using X‐tile plot, and participants were categorized accordingly. The Kaplan–Meier survival analysis was used to evaluate the relationship between these indicators and prognosis. Independent prognostic predictors of overall survival (OS) were identified using the multivariate Cox regression model.

**Results:**

This study included 214 patients diagnosed with NDMM with a median age of 61.7 ± 9.3 years, of whom 87 (40.7%) were female. Sarcopenia was identified in 80 patients (37.4%), with a higher prevalence in women (*p* = 0.015), those aged over 65 years (*p* = 0.013) and patients with a BMI ≤ 24 kg/m^2^ (*p* < 0.001). Patients with sarcopenia exhibited shorter progression‐free survival (PFS, *p* = 0.0497) and OS (*p* < 0.0001). Survival curves revealed that low PNI and high CONUT score were significantly correlated with adverse PFS and OS (all *p* < 0.05). A combined model incorporating sarcopenia, PNI and CONUT score demonstrated a strong association with PFS (*p* = 0.0007) and OS (*p* < 0.0001), and patients with concurrent sarcopenia, low PNI and high CONUT score exhibited the poorest survival rates. Multivariate Cox analysis indicated that sarcopenia was a significant and independent predictor of shorter OS (hazard ratio [HR] = 2.748, 95% confidence interval [CI]: 1.495–5.052, *p* = 0.001) in patients with NDMM.

**Conclusions:**

Sarcopenia is an independent prognostic indicator of OS in patients with NDMM. The integration of PNI and CONUT scores provides a comprehensive prognostic model for prognosis in patients with MM, highlighting the importance of functional status and nutritional assessments in clinical management.

## 1 Introduction

Multiple myeloma (MM) is an aggressive form of plasmacytoma that predominantly affects older individuals, with a mean age of approximately 69 years at diagnosis [1]. The prognosis of patients with MM has improved significantly with the development of novel therapeutic agents. However, MM remains incurable, with heterogeneous survival outcomes. Patients with high‐risk factors have poor survival outcomes. Disease‐related prognostic factors (such as cytogenetic abnormalities, circulating plasma cell proliferation and high lactic dehydrogenase) and treatment‐associated risk variables (such as depth of therapeutic remission or minimal residual disease) have been extensively studied and are routinely used in clinical practice. However, host‐specific characteristics, including body composition and nutritional status, have not received adequate attention. Due to the absence of simple and universal malnutrition screening tools, evaluation of nutritional status has not been conducted as standard practice in most cancer treatments. Therefore, it is essential to identify predictive biomarkers to accurately stratify patients with MM so that those at higher risk of poor prognosis can be detected early.

Sarcopenia, characterized by the loss of skeletal muscle mass and function, is prevalent in patients with cancer [2]. It is primarily caused by aging but can also result from inactivity, malnutrition and chronic diseases, such as cancers [3]. Sarcopenia is associated with increased chemotherapy toxicity, reduced compliance with oncological treatments [4] and diminished quality of life [5] in individuals with cancer. Previous studies have demonstrated the prognostic value of sarcopenia in predicting outcomes for patients with various malignancies [6]. CT images from whole‐body low‐dose CT and PETCT, routinely performed for the diagnosis and staging of MM, can also be used to identify sarcopenia by measuring the skeletal muscle areas at the level of the third lumbar (L3) vertebra from single cross‐sectional images. Previous studies have confirmed that L3 muscle areas correlate with total body muscle volume [7]. This reliable and non‐invasive imaging method enables the accurate assessment of patient body composition without additional costs or radiation exposure.

Malnutrition, defined as an imbalance between energy intake and expenditure, is common in patients with cancer, particularly in advanced stages. Accumulating evidence has shown that malnutrition is closely associated with treatment response and survival of patients with cancer [8]. Kim et al. [9] reported that the prevalence of malnutrition in patients with MM is approximately 70% and that malnourished patients with MM had poorer survival rates. The prognostic nutritional index (PNI) and controlling nutritional status (CONUT), both of which incorporate readily available laboratory parameters, are objective indices that reflect the nutritional and immune statuses of patients.

Recent studies have evaluated the prognostic significance of malnutrition indicators in patients with hematological malignancies [10, 11, 12]. However, their utility in MM remains unclear due to the limited number of available studies and contradictory findings. Given these limitations, a combination of multiple indicators may provide a more comprehensive reflection of patients' general status and facilitate improved patient stratification. Consequently, this study aimed to exploratively investigate the prognostic implications of sarcopenia, PNI and CONUT score in patients with NDMM.

## 2 Methods

### 2.1 Study Population

A total of 214 patients diagnosed with NDMM who underwent whole‐body low‐dose CT or PETCT scan at diagnosis between January 2016 and December 2022 at our institution were enrolled. This study adhered to the guidelines outlined in the Declaration of Helsinki and was approved by the Ethics Committee of the First Affiliated Hospital of Nanjing Medical University (Approval No. 2022‐SR‐448).

Clinical information was collected, including age, gender, BMI, the type of M protein, ISS stage, R‐ISS stage, bone lesions, extramedullary disease (EMD), hemoglobin (HB), the serum levels of lactate dehydrogenase (LDH) and albumin (ALB), the percentage of bone marrow plasma cells (BMPC), cytogenetics, the regimen used in the induction therapy and autologous stem cell transplantation (ASCT).

### 2.2 Assessment of Sarcopenia

To evaluate sarcopenia, skeletal muscle mass was assessed by measuring the skeletal muscle area at the L3 level with Hounsfield unit (HU) values between −29 and 150 using the low‐dose CT component of whole‐body CT or PET/CT scans acquired at diagnosis. The skeletal muscle index (SMI) was calculated using the following formula: SMI = L3 skeletal muscle area (cm^2^) / height squared (m^2^). The optimal cut‐off value for SMI was determined using the X‐tile software (https://medicine.yale.edu/lab/rimm/research/software/). At our centre, the thresholds for SMI were 45.1 cm^2^/m^2^ for men and 38.1 cm^2^/m^2^ for women, respectively. Patients with an SMI below the threshold were defined as sarcopenic.

### 2.3 Assessment of Nutritional Indices

Two nutritional indices were used to evaluate the nutritional status of participants. The PNI was calculated using the following equation: PNI = serum ALB (g/L) + 5 × peripheral blood lymphocyte count (×10^9^/L) [13]. The CONUT score was estimated using serum ALB, total cholesterol and peripheral blood lymphocyte counts as previously described [14]. The PNI and CONUT score were selected for this study as validated nutritional assessment tools that can be derived easily from routine laboratory parameters (serum ALB, lymphocyte count and total cholesterol). The thresholds for PNI and CONUT were 39.2 and 4, respectively, selected using X‐tile (https://medicine.yale.edu/lab/rimm/research/software/) as described above.

### 2.4 Survival Data

The last follow‐up was conducted in September 2023, with a median follow‐up period of 35 months. Progression‐free survival (PFS) was defined as the interval from diagnosis to disease progression or the final follow‐up. Overall survival (OS) was defined as the duration from diagnosis to death due to any cause or the final follow‐up.

### 2.5 Statistical Analysis

All data were analysed using SPSS Version 26.0 and Prism Version 10. Categorical variables are presented as frequencies and percentages, and the chi‐squared test was used to examine the clinical variables. Survival curves were constructed using the Kaplan–Meier method. A Cox proportional hazards regression model was used to identify factors that independently affected the survival of patients with MM. For all tests, statistical significance was set at *p* < 0.05.

## 3 Results

### 3.1 Patient Characteristics

This study included 127 (59.3%) males and 87 (40.7%) females. The median age of all the patients was 61.7 ± 9.3 years (range: 30–86 years). A total of 116 (56.0%) patients had a BMI of ≤ 24 kg/m^2^. The types of M protein included IgG (*n* = 97, 45.3%), IgA (*n* = 55, 25.7%), light chains (*n* = 45, 21.0%) and others (*n* = 17, 8.0%). Among the study population, 70.1% had bone lesions, while EMD was observed in 32.7% and high‐risk cytogenetic abnormalities in 27.7%. Regarding induction therapy, 59.9% of patients received proteasome inhibitors (PIs) in combination with immunomodulatory drugs (IMiDs), while 35.4% were treated with PIs alone. Only a small percentage (4.7%) of patients received IMiDs‐based therapy. In addition, 62 patients (29.0%) underwent autologous hematopoietic stem cell transplantation. The clinical characteristics of the patients are summarized in Table 1.

**TABLE 1:** Baseline characteristics.

		Total (*N* = 214)	With sarcopenia (*N* = 80, 37.4%)	Without sarcopenia (*N* = 134, 62.6%)	*p*
Age	≤ 65 years	130 (60.7)	40 (50.0)	90 (67.2)	**0.013**
	> 65 years	84 (39.3)	40 (50.0)	44 (32.8)	
Gender	Male	127 (59.3)	39 (48.8)	88 (65.7)	**0.015**
	Female	87 (40.7)	41 (51.2)	46 (34.3)	
BMI (kg/m^2^)	≤ 24	116 (56.0)	57 (73.1)	59 (45.7)	**< 0.001**
	> 24	91 (44.0)	21 (26.9)	70 (54.3)	
M protein	IgG	97 (45.3)	33 (41.2)	64 (47.8)	0.346
	IgA	55 (25.7)	18 (22.5)	37 (27.6)	
	Light chain	45 (21.0)	21 (26.3)	24 (17.9)	
	Others	17 (8.0)	8 (10.0)	9 (6.7)	
ISS stage	I	42 (19.6)	13 (16.3)	29 (21.6)	0.436
	II	84 (39.3)	30 (37.5)	54 (40.3)	
	III	88 (41.1)	37 (46.2)	51 (38.1)	
R‐ISS stage	I	27 (13.1)	9 (11.5)	18 (14.1)	0.703
	II	137 (66.5)	51 (65.4)	86 (67.2)	
	III	42 (20.4)	18 (23.1)	24 (18.7)	
Bone lesions	With	150 (70.1)	54 (67.5)	96 (71.6)	0.522
	Without	64 (29.9)	26 (32.5)	38 (28.4)	
EMD	With	70 (32.7)	28 (35.0)	42 (31.3)	0.581
	Without	144 (67.3)	52 (65.0)	92 (68.7)	
LDH (IU/L)	Normal	185 (86.4)	73 (91.2)	112 (83.6)	0.113
	Elevated	29 (13.6)	7 (8.8)	22 (16.4)	
HB (g/L)	< 100	126 (58.9)	48 (60.0)	78 (58.2)	0.797
	≥ 100	88 (41.1)	32 (40.0)	56 (41.8)	
ALB (g/L)	< 35	122 (57.0)	47 (58.7)	75 (56.0)	0.691
	≥ 35	92 (43.0)	33 (41.3)	59 (44.0)	
BMPC (%)	≤ 60	188 (89.5)	72 (92.3)	116 (87.9)	0.311
	> 60	22 (10.5)	6 (7.7)	16 (12.1)	
Cytogenetics	HRCA	51 (27.7)	20 (29.9)	31 (26.5)	0.625
	Without HRCA	133 (72.3)	47 (70.1)	86 (73.5)	
Induction therapy	PI‐based	75 (35.4)	30 (37.5)	45 (34.1)	0.072
	IMiDs‐based	10 (4.7)	7 (8.8)	3 (2.3)	
	PI + IMiDs	127 (59.9)	43 (53.7)	84 (63.6)	
ASCT	Yes	62 (29.0)	18 (22.5)	44 (32.8)	0.107
	No	152 (71.0)	62 (77.5)	90 (67.2)	

*Note:* Bold *p* value means *p* < 0.05.

Abbreviations: ALB: albumin, ASCT: autologous stem cell transplant, BMI: body mass index, BMPC: bone marrow plasma cells, EMD: extramedullary disease, HB: hemoglobin, HRCA: high‐risk cytogenetic abnormality, IMiDs: immunomodulatory drugs, ISS: international staging system, LDH: lactate dehydrogenase, PI: proteasome inhibitor, R‐ISS stage: revised international staging system.

**FIGURE 1:**
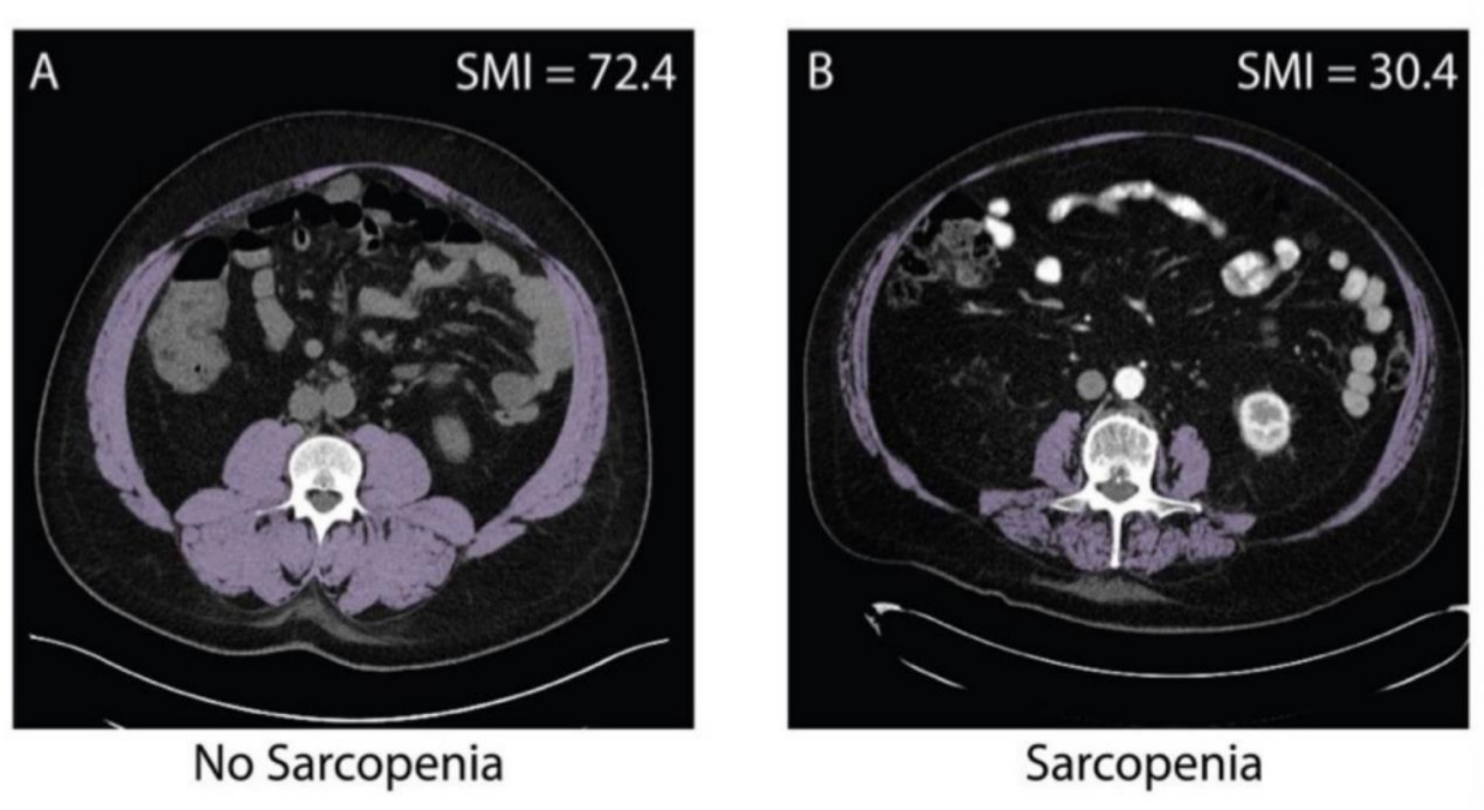
Representative examples of cross‐sectional computed tomography images at L3 level used for the assessment of skeletal muscle mass in (A) a non‐sarcopenic MM patient and (B) a sarcopenic MM patient.

Among the patients, 80 individuals (37.4%) were defined as sarcopenic, while 134 individuals (62.6%) were non‐sarcopenic, based on sex‐specific SMI cutoff. Figure 1 displays the skeletal muscle areas measured by CT, one from a patient with MM diagnosed with sarcopenia and the other from a patient without sarcopenia. The incidence of sarcopenia was significantly higher in patients aged > 65 years (*p* = 0.013), females (*p* = 0.015) and those with a lower BMI (*p* < 0.001). However, no significant differences were observed in tumour burden or treatment regimens between patients with or without sarcopenia (Table 1).

### 3.2 Survival Analysis of Patients With Sarcopenia

**FIGURE 2:**
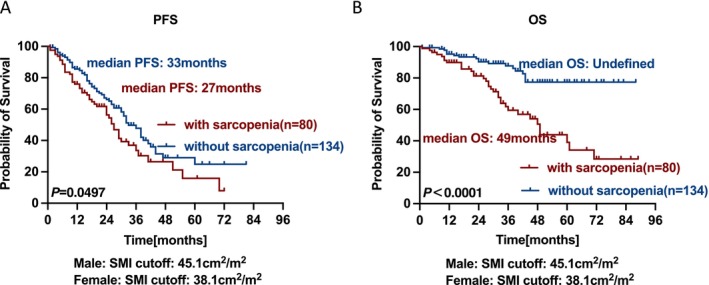
PFS (A) and OS (B) survival curves in NDMM patients with and without sarcopenia.

The median follow‐up period was 35 months. By the end of follow‐up, 112 patients had experienced disease progression, and 51 patients had died. Compared with non‐sarcopenic individuals, those with sarcopenia had significantly shorter PFS (median PFS: 27 months vs. 33months, *p* = 0.0497) and OS (median OS: 49 months vs. undefined, *p* < 0.0001) (Figure 2A,B). Among patients without sarcopenia, the 5‐year PFS rate was 24.9%, compared with a 15.9% rate in the group with sarcopenia. Similarly, the 5‐year OS rates were 77.3% and 39.0% in the non‐sarcopenic and sarcopenic groups, respectively.

### 3.3 Survival Analysis of Patients With Malnutrition

**FIGURE 3:**
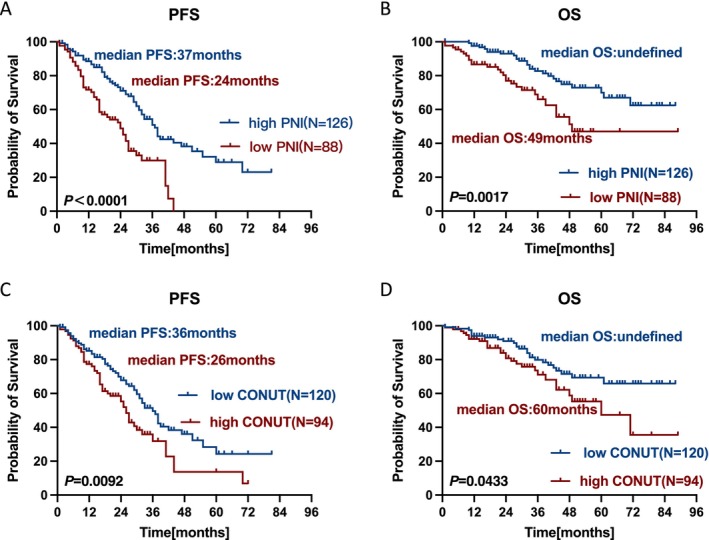
Kaplan–Meier curves of patients stratified by PNI and CONUT score. PFS (A) and OS (B) survival curves in patients with low PNI and high PNI; PFS (C) and OS (D) survival curves in patients with low CONUT score and high CONUT score.

To evaluate the predictive ability of the nutrition screening tools, patients were divided into groups based on PNI and CONUT scores. The low PNI group comprised 88 patients (41.1%), while the high PNI group enrolled 126 patients (58.9%). The low and high CONUT groups consisted of 120 (56.1%) and 94 (43.9%) patients, respectively. Survival curves indicated that patients with low PNI had worse PFS (median PFS: 24 months vs. 37 months, *p* < 0.0001) and OS (median OS: 49 months vs. undefined, *p* = 0.0017) than those with high PNI (Figure 3A,B). In addition, high CONUT score was associated with poorer PFS (median PFS: 26 months vs. 36 months, *p* = 0.0092) and OS (median OS: 60 months vs. undefined, *p* = 0.0433) (Figure 3C,D).

### 3.4 Combination of Sarcopenia, PNI, and CONUT as a Prognostic Marker for NDMM Patients

**FIGURE 4:**
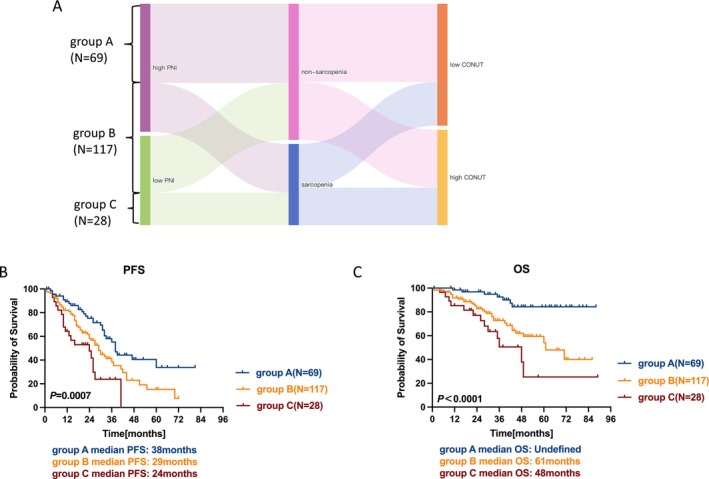
The Sankey diagram visualized the classification process based on the thresholds of sarcopenia, PNI and CONUT score (A). Kaplan–Meier curves of PFS (B) and OS (C) of the patients with NDMM stratified by the combination of sarcopenia, PNI and CONUT score.

Patients were divided into three groups based on their sarcopenia status; PNI and CONUT score to further explore the combined effect of these factors on MM prognosis. Group A included patients with high PNI, non‐sarcopenia and low CONUT score (*n* = 69), whereas Group C included patients with low PNI, sarcopenia and high CONUT (*n* = 28). The remaining patients were classified into Group B (*n* = 117). The Sankey diagram shown in Figure 4A clearly illustrates this classification in detail. Kaplan–Meier survival curves revealed significant differences in PFS rates among these three groups (*p* = 0.0007), with the lowest rate observed in Group C and the highest rate in Group A (Figure 4B). The OS curves showed similar results (*p* < 0.0001) (Figure 4C). Survival analysis suggested that sarcopenic individuals with coexisting low PNI and high CONUT scores had the worst prognosis.

### 3.5 Univariate and Multivariate Analysis of Predictive Factors of the OS of Patients With NDMM

Viables including BMI, sarcopenia, PNI and CONUT score were incorporated into the univariate analysis to investigate their predictive capability for MM. Univariate analysis illustrated that sarcopenia (HR = 3.255, 95% CI: 1.885–5.712, *p* < 0.001), BMI > 24 kg/m^2^ (HR = 0.479, 95% CI: 0.316–0.726, *p* = 0.021), low PNI (HR = 2.347, 95% CI: 1.354–4.068, *p* = 0.012) and high CONUT (HR = 1.733, 95% CI: 1.008–2.978, *p* = 0.047) were significantly correlated with OS. Those variables were selected and sequentially assessed using multivariate analysis. Multivariate analysis revealed that sarcopenia (HR = 2.748, 95% CI: 1.495–5.052, *p* = 0.001) was the only independent prognostic indicator for OS (Table 2).

**TABLE 2:** Univariate and multivariate analysis for OS of the patients with MM.

	Univariate analysis		Multivariate analysis	
	HR (95% CI)	*p*	HR (95% CI)	*p*
With sarcopenia	3.255 (1.855–5.712)	**< 0.001**	2.748 (1.495–5.052)	**0.001**
BMI > 24 kg/m^2^	0.479 (0.316–0.726)	**0.021**	0.575 (0.368–0.899)	0.055
Low PNI	2.347 (1.354–4.068)	**0.012**	2.549 (1.130–5.749)	0.054
High CONUT	1.733 (1.008–2.978)	**0.047**	0.760 (0.341–1.696)	0.503

*Note:* Bold *p* value means *p* < 0.05.

Abbreviations: BMI: body mass index, CI: confidence interval, CONUT: controlling nutritional status, HR: hazard ratio, PNI: prognostic nutritional index.

## 4 Discussion

MM is a highly heterogeneous disease, and treatment strategies should be tailored not only to cancer characteristics but also to host‐specific factors. Nutritional status is a crucial host factor that influences the prognosis of patients with cancer [15]. This study aimed to investigate the prognostic potential of sarcopenia and nutritional parameters in patients with MM. We found that sarcopenia, low PNI and high CONUT score were all correlated with poor survival, and that sarcopenia was an independent prognostic indicator of OS in patients with NDMM. Furthermore, the combination of sarcopenia, PNI and CONUT score established a comprehensive model for predicting survival outcomes in patients with MM. This prognostic model could be a simple and feasible screening tool for detecting prognosis in patients with MM and may help guide treatment decisions.

Sarcopenia, a progressive skeletal muscle disorder characterized by decreased muscle strength and mass, with or without impaired physical performance [3] has been linked to impaired tolerance to various cancer treatments and adverse prognosis [16, 17]. Diagnosis of sarcopenia may be based on imaging techniques including CT/MRI, dual‐energy X‐ray absorptiometry (DEXA), bioimpedance analysis (BIA) and ultrasonography [18]. MM often presents with clinical manifestations involving osteolytic bone lesions and EMD. In clinical practice, whole‐body low‐dose CT is routinely used to assess bone lesions, and PETCT is employed to evaluate both bone lesions and EMD. Therefore, our study utilized the low‐dose CT component of whole‐body CT or PET/CT as the method for assessing sarcopenia. Considering that the currently accepted thresholds are based on muscle mass data from different continents (Europe, Asia, America), we established optimal SMI cut‐off value based on the X‐tile software. In this study, sarcopenia was observed in 37.4% of the participants and was associated with advanced age, female gender and lower BMI. It served as a marker of inferior survival and a stand‐alone prognostic marker for OS in patients with NDMM. These findings align with those of Nandakumar et al. [19], who used a deep learning algorithm to measure muscle mass at the L3 level from CT images of 322 patients with NDMM, demonstrating that sarcopenia was associated with adverse OS and was an independent risk factor. However, most previous studies have not found a significant association between sarcopenia and survival in MM patients [20, 21, 22], which appears to contradict the findings of this study. For example, Abdallah et al. reported that sarcopenia was not linked to survival outcomes by measuring muscle mass or muscle quality [22]. These inconsistent results may be attributed to the different methods of evaluating sarcopenia, including varying imaging techniques, skeletal muscle sites and cutoff values.

Patients with cancer are particularly susceptible to malnutrition and disease‐associated catabolic derangements. Various nutritional indices have been used to evaluate patients' nutritional status and assist in clinical risk stratification. The PNI and CONUT score, which can be easily derived from routine laboratory tests, reflect patient malnutrition and systemic inflammation status. In the present study, PNI and CONUT score were significantly associated with OS but did not demonstrate independent prognostic value. Both indices incorporate serum ALB levels and lymphocyte count. ALB has been identified as a significant predictor of hematological malignancies [23] and is also a key parameter of the ISS [24]. Lymphocytes play a crucial role in inhibiting the growth and spread of malignancies and could reflect host immune status [25]. The PNI, based on these two biomarkers, has proven effective in predicting the prognosis in various solid tumours and hematological malignancies [26, 27]. However, its prognostic value in MM remains unclear. Liang et al. conducted a retrospective experiment to explore the predictive capability of PNI among 157 NDMM patients [28] and found that a low PNI was correlated with poorer prognosis.

Compared with the PNI, the CONUT score also incorporates total cholesterol levels into its calculation. Hypocholesterolemia has been observed in patients with MM, and cholesterol levels are lower in advanced stages [29]. Several studies have investigated the predictive role of the CONUT score in MM. Liang et al. found that the CONUT score was closely correlated with OS but was not retained as an independent prognostic factor in patients with NDMM, consistent with our findings of this study [28]. However, most studies have demonstrated that a high CONUT score is an independent risk factor for prognosis in individuals with MM [30, 31, 32]. These discrepancies may be attributed to the heterogeneity in patient populations and the thresholds for nutritional indices. Further studies are needed to explore the prognostic role of the PNI and CONUT score in MM.

As sarcopenia is a progressive skeletal muscle disorder, its development is relatively slow. In contrast, blood biomarkers are more sensitive to changes in nutritional status and can promptly reflect a patient's condition. The incorporation of nutritional indices with sarcopenia may provide a more comprehensive reflection of a patient's general status and accurately predict the clinical outcomes of patients with MM. Our findings revealed that this integrative approach was effective in stratifying patients with NDMM, and that patients with sarcopenia, low PNI and high CONUT score concurrently had the worst prognosis. SMI reflects muscle mass, while PNI assesses inflammatory or immunosuppressive signals not reflected by SMI. CONUT additionally includes information related to lipid metabolism. Although the multivariate analysis indicated that only SMI is an independent prognostic risk factor, the amalgamation of SMI, PNI and CONUT score assesses patient status from the perspectives of muscle mass, immunity and metabolic function, respectively. This integration allows for a more personalized and targeted approach to malnutrition management, such as physical exercise and nutritional supplementation, which may enhance treatment efficacy and improve the prognosis of patients.

## 5 Conclusion

This retrospective exploratory study demonstrated that sarcopenia is an independent prognostic factor for OS in patients with NDMM. The integration of sarcopenia with the PNI and CONUT score provided a comprehensive predictive model for assessing prognosis in these patients. Malnutrition screening in patients with MM is valuable for risk stratification and individualized treatment strategies. Further investigations are warranted to validate these findings and enhance our understanding of the role of sarcopenia in the progression and management of MM.

## Funding

This work was supported by the National Natural Science Foundation of China (No. 82200223, No. 82370205 and No. 82070223) and the Social Development Project of Jiangsu Science and Technology Plan (No. BK20220718).

## Conflicts of Interest

The authors declare no conflicts of interest.
